# CT perfusion for predicting intracranial atherosclerotic middle cerebral artery occlusion

**DOI:** 10.3389/fneur.2024.1406224

**Published:** 2024-06-21

**Authors:** Zigao Wang, Abudoukeyoumu Yasheng, Yifeng Ling, Hongchen Zhao, Yiting Mao, Shilin Yang, Wenjie Cao

**Affiliations:** ^1^Department of Neurology and Institute of Neurology, Huashan Hospital, Fudan University, Shanghai, China; ^2^Department of Neurology, Kashi Prefecture Second People’s Hospital, Kashi, China

**Keywords:** middle cerebral artery occlusion, intracranial atherosclerotic stenosis, embolism, computed tomography perfusion, core volume growth rate

## Abstract

**Backgrounds and purpose:**

Identifying the underlying cause of acute middle cerebral artery occlusion (MCAO) as intracranial atherosclerotic stenosis (ICAS) or embolism is essential for determining the optimal treatment strategy before endovascular thrombectomy. We aimed to evaluate whether baseline computed tomography perfusion (CTP) characteristics could differentiate ICAS-related MCAO from embolic MCAO.

**Methods:**

We conducted a retrospective analysis of the clinical and baseline CTP data from patients who underwent endovascular thrombectomy for acute MCAO between January 2018 and December 2022. Core volume growth rate was defined as core volume on CTP divided by onset to CTP time. Multivariate logistic analysis was utilized to identify independent predictors for ICAS-related acute MCAO, and the diagnostic performance of these predictors was evaluated using receiver operating characteristic curve analysis.

**Results:**

Among the 97 patients included (median age, 71 years; 60% male), 31 (32%) were diagnosed with ICAS-related MCAO, and 66 (68%) had embolism-related MCAO. The ICAS group was younger (*p* = 0.002), had a higher proportion of males (*p* = 0.04) and smokers (*p* = 0.001), a lower prevalence of atrial fibrillation (AF) (*p* < 0.001), lower NIHSS score at admission (*p* = 0.04), smaller core volume (*p* < 0.001), slower core volume growth rate (*p* < 0.001), and more frequent core located deep in the brain (*p* < 0.001) compared to the embolism group. Multivariate logistic analysis identified core volume growth rate (aOR 0.46, 95% CI 0.26–0.83, *p* = 0.01) as an independent predictor of ICAS-related MCAO. A cutoff value of 2.5 mL/h for core volume growth rate in predicting ICAS-related MCAO was determined from the receiver operating characteristic curve analysis, with a sensitivity of 81%, specificity of 80%, positive predictive value of 66%, and negative predictive value of 90%.

**Conclusion:**

Slow core volume growth rate identified on baseline CTP can predict ICAS-related MCAO. Further prospective studies are warranted to confirm and validate these findings.

## 1 Introduction

Endovascular treatment (EVT) has become the standard of care for stroke resulting from acute large vessel occlusion (LVO) (1–6). The choice of optimal EVT strategy for acute LVO is influenced by the underlying causes, which primarily comprise embolism with a cardiac or arterial origin and *in-situ* thrombosis due to intracranial atherosclerotic stenosis (ICAS), a condition more prevalent among Asian patients (7, 8). While thrombectomy using a stent retriever and/or contact aspiration is effective for removing cardiac or arterial emboli, managing ICAS-related LVO often requires remedial therapies such as aggressive antiplatelet therapies, balloon angioplasty, and stent deployment (7, 9–11). Recently, a novel technique called BASIS, which is short for balloon angioplasty with the distal protection of stent retriever, has been demonstrated to be feasible and safe for treating ICAS-related LVO (12). Therefore, accurately identifying ICAS as the underlying cause of acute LVO before initiating EVT is essential for selecting the most suitable therapeutic approach.

Middle cerebral artery occlusion (MCAO) is responsible for more than 70% of acute large vessel occlusion (LVO) in the anterior circulation (1–6). Various preoperative neuroimaging markers have been proposed to distinguish ICAS from embolism as the cause of acute MCAO, including negative susceptibility vessel sign on T2*-weighted gradient-echo MR imaging and deep location of the infarct core on diffusion-weighted image (DWI) (13, 14). However, these predictors rely on MR imaging, which may not always be readily available in the emergency situations. In contrast, CT perfusion (CTP) is widely accessible and routinely performed for suspected LVO cases worldwide (15, 16). Till now, studies investigating the use of CTP in distinguishing ICAS from embolism as the cause of acute MCAO are still scarce. Hence, the present study was conducted to evaluate the effectiveness of the baseline CTP imaging characteristics in identifying ICAS-related acute MCAO.

## 2 Materials and methods

### 2.1 Study design and participants

This was a retrospective study conducted with ethical approval from the institutional review board of our institution and informed consent of the patients or their next of kin. Data was extracted from our prospective endovascular database to identify patients who had undergone endovascular thrombectomy for acute ischemic stroke due to LVO between January 2018 and December 2022. Inclusion criteria consisted of patients who met the following conditions: (1) onset of ischemic stroke within 24 h; (2) the stroke was due to proximal MCAO (M1 or M2 segment) confirmed by digital subtraction angiography (DSA); (3) CTP evaluation performed before EVT. Main exclusion criteria included: (1) bilateral MCAO; (2) unsuccessful recanalization of the occluded MCA; (3) evidence of Moyamoya disease, vasculitis, or dissection based on history and imaging findings.

### 2.2 Data collection

Clinical data, including demographics, comorbidities, National Institutes of Health Stroke Scale (NIHSS) score at admission, and electrocardiography results were extracted from our electronic medical records system.

Baseline computed tomography (CT), CT angiography, and digital subtraction angiography (DSA) were reviewed retrospectively by two experienced neurologists who were blinded to patients’ clinical information and CTP parameters. In cases of discrepancies between the reviewers, a third reviewer was assigned to make the final decision. The stenosis degree of the ipsilateral internal carotid artery (ICA) was assessed by CTA according to the North American Symptomatic Carotid Endarterectomy Trial (NASCET) criteria. The collaterals were evaluated by DSA using American Society of Interventional and Therapeutic Neuroradiology Collateral Grading (ACG) system. Good preprocedural collateral was defined as an ACG score ≥ 3 (17).

### 2.3 CTP imaging analysis

CTP evaluation was performed using Mistar (Apollo Medical Imaging Technologies, Australia) as described previously (18, 19). Hypoperfusion areas were defined as regions with a delay time > 3 s, while the core was defined as an area with relative cerebral blood flow <30%. Core volume growth rate was calculated by core volume divided by onset to CTP time, as described in previous studies (20, 21). The penumbra was measured by the hypoperfusion volume minus the core volume. The core located deep in the brain referred to the core located in the centrum semiovale or the basal ganglia, without involving the cerebral cortex.

### 2.4 Definitions of ICAS-related and embolic MCAO

**Figure 1:**
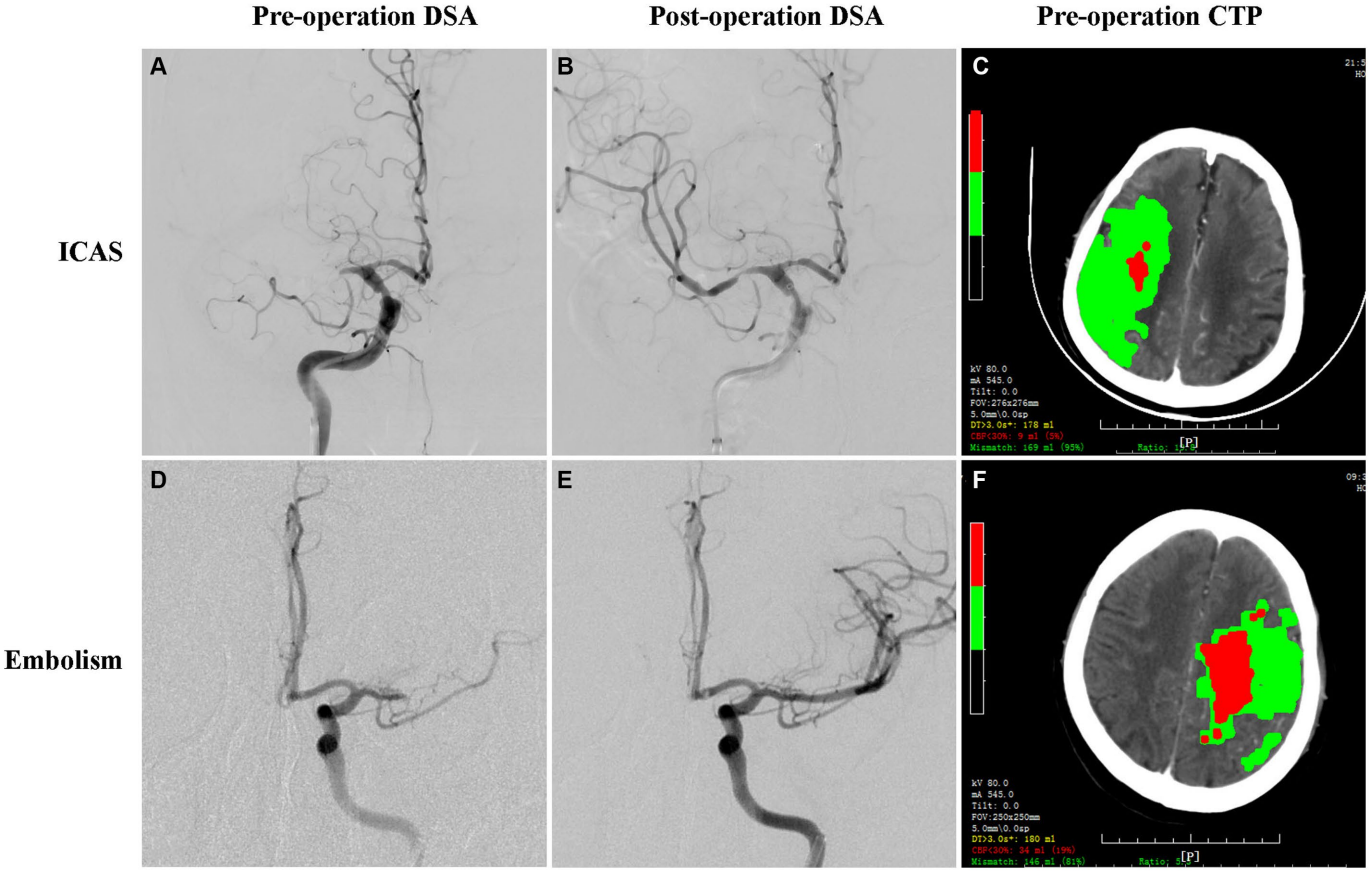
Illustrated images showing the catheter angiography and CTP maps of patients with acute MCAO due to ICAS and embolism. Angiography and baseline CTP of ICAS-related MCAO **(A–C)** and embolism-related MCAO **(D–F)**. Catheter angiography showing MCA occlusion before thrombectomy **(A,D)**, *in-situ* severe residual stenosis **(B)**, and complete recanalization **(E)** after thrombectomy with a stent retriever. Baseline CTP showing the cores (red), and the penumbras (green) **(C,F)**.

Modified from previous studies (22–24), the cause of the MCAO was categorized as ICAS if fixed stenosis (residual stenosis degree ≥70% or ≥50% with impaired antegrade flow) at the occlusion site or evidence of re-occlusion after mechanical thrombectomy was observed. On the other hand, it was classified as embolism if no evidence of focal stenosis was found after thrombectomy using a stent retriever and/or contact aspiration (Figure 1). The residual stenosis degree was assessed according to the Warfarin Aspirin Symptomatic Intracranial Disease (WASID) criteria.

### 2.5 Statistical analysis

Statistical analysis was performed using SPSS for Windows (version 25.0, IBM, United States). Categorical variables were reported as number (percentage), while continuous variables were reported as mean (SD) or median (interquartile range, IQR). The normality of distributions was assessed using the Shapiro–Wilk test and histograms. Categorical variables were analyzed using chi-square or Fisher exact tests, and continuous variables were analyzed using Student’s *t*-test or Mann–Whitney U test. Variables with a *p*-value < 0.1 in the univariate logistic regression analysis were included in the multivariate logistic regression analysis. Receiver operating characteristic (ROC) analysis was utilized to evaluate the predictive value of independent predictors for identifying ICAS-related MCAO by determining the area under the curve (AUC). The cut-off value was determined by maximizing the sum of sensitivity and specificity. All hypothesis testing was carried out using two-sided statistical tests, and *p* < 0.05 was considered statistically significant.

## 3 Results

### 3.1 Baseline clinical and CTP characteristics

A total of 97 eligible patients were included in the analysis. The median age was 71 (IQR, 60–79) years, with males accounting for 60% of the cohort. Hypertension was the most common comorbidity (64%), followed by atrial fibrillation (AF) (43%). The median NIHSS score at admission was 15 (IQR, 11–18). The occlusion was located at the M1 segment in 84% of these patients. The median volumes of the hypoperfusion area, ischemic core, and penumbra were 105 mL, 14 mL, and 79 mL, respectively (Table 1).

**Table 1:** Baseline clinical and CTP characteristics of patients with acute MCAO.

	All (*n* = 97)	ICAS (*n* = 31)	Embolism (*n* = 66)	*p* value
Age (year), median (IQR)	71 (60–79)	62 (55–71)	73 (66–81)	0.002
Male gender, *n* (%)	58 (60)	23 (74)	35 (53)	0.04
Hypertension, *n* (%)	62 (64)	22 (71)	40 (61)	0.32
Diabetes mellitus, *n* (%)	24 (25)	8 (26)	16 (24)	0.87
Dyslipidemia, *n* (%)	17 (18)	8 (26)	9 (14)	0.14
Smoking, *n* (%)	30 (31)	17 (55)	13 (19)	0.001
Coronary artery disease, *n* (%)	10 (10)	1 (3)	9 (14)	0.16
Atrial fibrillation, *n* (%)	42 (43)	3 (10)	39 (59)	<0.001
NIHSS, median (IQR)	15 (11–18)	12 (8–18)	15 (13–18)	0.04
Occlusion site, *n* (%)				0.51
M1 segment	81 (84)	27 (87)	54 (82)	
M2 segment	16 (17)	4 (13)	12 (18)	
Good collaterals, *n* (%)	27 (28)	14 (45)	13 (20)	0.04
Ipsilateral ICA stenosis ≥ 50%	15 (16)	8 (26)	7 (11)	0.07
Hypoperfusion volume (ml), median (IQR)	105 (62–147)	105 (56–150)	106 (63–145)	0.94
Core volume (ml), median (IQR)	14 (7–32)	5 (2–13)	23 (11–44)	<0.001
Core volume growth rate (ml/h), median (IQR)	3.8 (1.1, 11.0)	0.9 (0.1, 2.1)	6.0 (2.8, 19.5)	<0.001
Core located deep in the brain, *n* (%)	54 (59)	24 (89)	30 (47)	<0.001
Penumbra volume (ml), median (IQR)	79 (41–115)	101 (49–127)	69 (40–105)	0.05

Among these 97 patients, 31 (32%) had underlying ICAS (ICAS group), while 66 (68%) were diagnosed with embolism (embolism group). The ICAS group was younger (62 years vs. 73 years, *p* = 0.002), had a higher proportion of males (74% vs. 53%, *p* = 0.04) and smokers (55% vs. 19%, *p* = 0.001). They also a lower prevalence of atrial fibrillation (10% vs. 59%, *p* < 0.001), and a lower median NIHSS score at admission (12 vs. 15, *p* = 0.04) compared with the embolism group. Additionally, the ICAS group had a higher proportion of good leptomeningeal collaterals (45% vs. 20%, *p* = 0.04), smaller core volume (5 mL vs. 23 mL, *p* < 0.001), slower core volume growth rate (0.9 mL/h vs. 6 mL/h, *p* < 0.001), and a higher prevalence of core located deep in the brain (89% vs. 47%, *p* < 0.001) than the embolism group. No significant differences with regard to hypertension, diabetes, dyslipidemia, coronary disease, occlusion site of MCA, ipsilateral ICA stenosis, hypoperfusion volume, and penumbra volume were found between the two groups (Table 1).

### 3.2 Predictors for ICAS-related acute MCAO

After adjusting for potential confounding factors, including age, gender, history of smoking, absence of AF, NIHSS score on admission, collaterals, core volume growth rate, penumbra volume, and core location, multivariate logistic regression analysis identified core volume growth rate (aOR 0.46, 95% CI 0.26–0.83, *p* = 0.01) as an independent predictor of ICAS-MCAO (Table 2).

**Table 2:** Predictors for ICAS-MCAO by univariate and multivariate analysis.

Variables	Univariate		Multivariate	
	OR (95% CI)	*p* value	OR (95% CI)	*p* value
Age	0.96 (0.93–0.99)	0.01	0.95 (0.88–1.03)	0.20
Male	2.54 (0.99–6.51)	0.05	1.75 (0.22–14.04)	0.60
Smoking	4.95 (1.95–12.57)	0.001	1.02 (0.13–8.01)	0.98
Absence of AF	13.48 (3.72–48.87)	<0.001	5.87 (0.84–40.97)	0.07
NIHSS	0.92 (0.85–1.00)	0.05	1.10 (0.95–1.28)	0.20
Good collaterals	3.36 (1.32–8.53)	0.01	4.70 (0.67–33.03)	0.12
Penumbra volume	1.01 (1.00–1.02)	0.03	1.01 (0.99–1.03)	0.27
Core volume growth rate	0.58 (0.43–0.78)	<0.001	0.46 (0.26–0.83)	0.01
Core located deep in the brain	9.07(2.48–33.16)	0.001	9.82 (0.81–118.71)	0.07

### 3.3 Diagnostic performance of core volume growth rate

**Figure 2:**
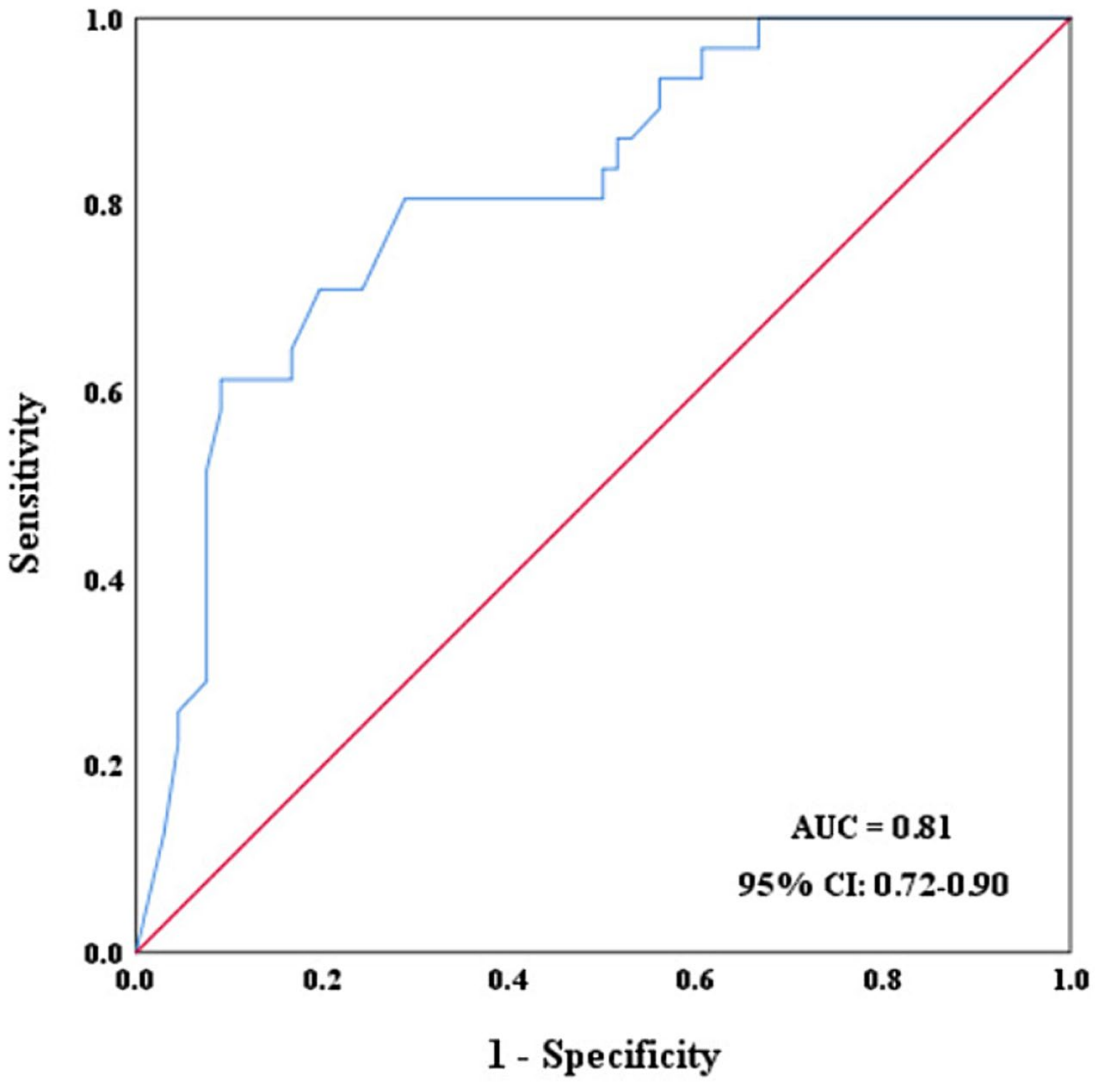
ROC curves of core volume growth rate for identifying ICAS-related MCAO.

ROC analysis showed that the area under the curve (AUC) for core volume growth rate in identifying ICAS-related MCAO was 0.86 (95% CI: 0.79–0.94) (Figure 2). The optimal cut-off value for core volume growth rate, which yielded the highest sensitivity and specificity, was determined to be 2.5 mL/h, with a sensitivity of 81%, specificity of 80%, positive predictive value of 66%, and negative predictive values of 90%, respectively (Table 3).

**Table 3:** Diagnosis performance of core volume growth rate <2.5 ml/h for predication ICASL-MCAO.

Sensitivity (95% CI)	Specificity (95% CI)	PPV (95% CI)	NPV (95% CI)
0.81 (0.63–0.93)	0.80 (0.69–0.89)	0.66 (0.49–0.80)	0.90 (0.79–0.96)

## 4 Discussion

In our present study, we found that ICAS accounted for nearly one-third of acute MCAO cases. Importantly, core volume grow rate identified by baseline CTP was an independent predictor of ICAS-related acute MCAO. A core volume grow rate slower than 2.5 mL/h could help distinguish ICAS from embolism as the etiology of acute MACO.

ICAS is a prominent cause of LVO globally, particularly in Asia. However, accurately identification of ICAS-related LVO can be challenging, requiring neuropathological evidence of atherosclerotic plaques within the occluded vessel wall. In clinical practice, neuroimaging, including catheter angiography, plays a critical role in diagnosis of ICAS-related LVO. Residual fixed stenosis greater than 50% and stenosis with re-occlusion on follow-up angiography are commonly used criteria to define ICAS-related LVO (24). Our present study modified the definition of ICAS-MCAO and found that 32% of Chinese patients with acute MCAO had underlying ICAS, which was in agreement with previous studies reporting rates ranging from 7 to 44% (2, 25–27).

Preoperative identification of underlying ICAS significantly influences therapeutic strategies and patients’ prognosis in LVO cases. Previous study has shown that using a stent retriever as the first-line EVT device for ICAS-rerated LVO leads to a higher successful reperfusion rate, fewer rescue treatments, and reduced perioperative complications compared to contact aspiration (28). A *post hoc* analysis of the Endovascular Treatment With versus Without Tirofiban for Patients with Large Vessel Occlusion Stroke (RESCUE BT) trial demonstrated that tirofiban administration before EVT significantly improved the functional independence at 90 days without increasing symptomatic intracranial hemorrhage in patients with ICAS-related LVO (29). These findings suggest that early identification of ICAS as the underlying cause of vessel occlusion is essential for choosing appropriate EVT modality to achieve a faster recanalization, a lower complication, and a better clinical outcome for patients with stroke due to acute LVO.

Herein, we demonstrated that baseline CTP characteristics could help distinguish ICAS from embolism as the cause of MCAO. Patients with ICAS-related MCAO exhibited different ischemic core characteristics compared with those with embolic MCAO, including smaller core volume and core location more likely in the deep brain. In agreement with our findings, previous studies have also demonstrated that the baseline core volume is larger in patients with embolic LVO than in those with non-embolic LVO (22, 30). Meanwhile, previous research using DWI to evaluate the infarct core in patients with acute MCAO also found that the ICAS group was more likely to have an infarct core in the deep part of the brain than the embolism group (14). Most importantly, we further revealed that core volume growth rate could independently predict ICAS-MCAO with an adjusted odds ratio of 0.46. Consistently, a previous study using RAPID software to evaluate cerebral perfusion state showed that core volume growth rate was also significantly lower in patients with ICAS-related LVO than that in those with embolism-related LVO (31). These data suggest that patients with acute MCAO who exhibit slower core volume growth rate are more likely to have underlying ICAS.

The exact mechanisms underlying the smaller core volume, slower core growth rate, and core located deep in the brain observed in ICAS-related MCAO are still unclear. It has been suggested that the collaterals within hypoperfusion brain regions play an important role in determining the fate of these tissues (32). Better collaterals are often associated with smaller infarct cores and milder clinical symptoms (33–35). Better collateral flow has also been demonstrated to be associated with slower core growth rate (20). Interestingly, previous studies had reported that good leptomeningeal collaterals are more common in patients with ICAS-related LVO compared with those with embolic LVO. For example, Yi et al. reported better meningeal collaterals demonstrated by DSA in patients with ICAS-related LVO compared with those with embolic LVO (36). Another study using CTA also showed that good leptomeningeal collaterals were more frequently seen in patients with ICAS-related LVO than in those with embolic LVO (37). In consistent with these studies, we demonstrated in the present study that the ICAS group had better collaterals than did the embolism group. Therefore, it is plausible to infer that the development of good leptomeningeal collaterals in patients with ICAS-related MCAO may slow down the core growth rate and protect them from developing large territorial infarctions when they experience acute occlusion of the stenotic MCA. Meanwhile, the better leptomeningeal collaterals make cortical neurons more resistant to acute ischemic insult than those deep in the brain. Therefore, when the stenotic MCA occludes, neuronal death is more likely to occur deep in the brain rather than in the cortex, resulting in the deeper distribution of the infarct core.

Patients with AF have a three to five-fold increased risk of cerebral embolism (38), which indicates that LVO in patients with a history of AF is more likely to result from embolism. However, it does not mean that a history of AF can rule out the possibility of ICAS as the underlying cause of acute MCAO, given that a substantial proportion of patients with underlying ICAS may also have co-existing AF. Previous studies reported a rate of a history of AF in non-embolic LVO stroke patients ranging from 5 to 42.9% (13, 14, 23, 39). In agreement with these findings, our present study demonstrated that AF was found in 10% of patients with ICAS-related MCAO. Therefore, ICAS should also not be excluded as the underlying cause of MCAO in patients with a history of AF, especially in those exhibiting small core volume and slow core growth rate.

Several limitations are present in this study. Firstly, this was a retrospective study lacking randomization, which might carry a high risk of selection bias. Secondly, the sample size was relatively small, which might limit the statistical power of the analysis. Thirdly, only patients with MCAO were included, limiting the generalizability of the findings to other cerebral artery occlusions. Fourthly, the study only included Chinese patients, limiting the generalizability of the results to other ethnicities. Future prospective studies with larger and more diverse populations are needed to validate our findings.

## 5 Conclusion

In conclusion, our study identified that a slow core volume growth rate was an independent predictor of ICAS-related acute MCAO, suggesting that baseline CTP characteristics can aid in distinguishing ICAS from embolism as the cause of MCAO. Further prospective studies with larger sample sizes are necessary to confirmed and validate these findings.

## Data availability statement

The raw data supporting the conclusions of this article will be made available by the authors, without undue reservation.

## Ethics statement

The studies involving humans were approved by the Institutional Review Board of Huashan Hospital, Fudan University, China. The studies were conducted in accordance with the local legislation and institutional requirements. The participants provided their written informed consent to participate in this study.

## Author contributions

ZW: Conceptualization, Data curation, Funding acquisition, Investigation, Methodology, Validation, Writing – original draft, Writing – review & editing. AY: Conceptualization, Formal analysis, Investigation, Methodology, Writing – original draft. YL: Conceptualization, Data curation, Formal analysis, Supervision, Validation, Visualization, Writing – original draft. HZ: Conceptualization, Methodology, Project administration, Resources, Validation, Writing – original draft. YM: Conceptualization, Data curation, Formal analysis, Software, Supervision, Validation, Writing – review & editing. SY: Conceptualization, Data curation, Formal analysis, Investigation, Methodology, Project administration, Validation, Writing – review & editing. WC: Formal analysis, Resources, Supervision, Validation, Visualization, Writing – original draft, Writing – review & editing.
